# Retrospective Analysis of 28 Cases of Tuberculosis in Pregnant Women in China

**DOI:** 10.1038/s41598-019-51695-8

**Published:** 2019-10-25

**Authors:** Qiang Li, Yanhua Song, Hongmei Chen, Li Xie, Mengqiu Gao, Liping Ma, Yinxia Huang

**Affiliations:** 1 Department of Tuberculosis, Beijing Chest Hospital, Capital Medical University, Beijing Tuberculosis and Thoracic Tumor Research Institute, Beijing, China; 2 Beijing Key Laboratory for Drug Resistant Tuberculosis Research, Beijing Tuberculosis and Thoracic Tumor Research Institute, Beijing Chest Hospital, Capital Medical University, Beijing, China; 3 Department of Infectious Diseases, Capital Medical University, Beijing, China

**Keywords:** Tuberculosis, Tuberculosis, Epidemiology, Epidemiology

## Abstract

While tuberculosis (TB) in pregnant women is reported globally, clinical data is unavailable in China. To describe clinical features and identify difficulties in the diagnosis of pregnancy-related TB, we performed a retrospective study of 28 TB inpatients at Beijing Chest Hospital. The results were presented in terms of interquartile range (IQR) for age, and medians and percentages with respect to the categorical variables. One patient (3.6%) was immediately diagnosed; for 27 patients (96.4%), the median interval from the initial onset of symptoms to diagnosis was five weeks. Eight cases (28.6%) were microbiologically confirmed. 22 (78.6%) were pulmonary TB (PTB), while six (21.4%) were extrapulmonary TB (EPTB). In addition, eight (28.6%) were miliary TB and six (21.4%) were cerebral TB. 27 (96.4%) were cured and one (3.6%) died. 15 neonates were identified, nine of which were healthy. Two were small for the gestational age (SGA) and one was a stillbirth. Three had neonatal TB, one of which died. Nine were legal abortions and four were spontaneous abortions. Indeed, there was a substantial delay in the diagnosis of TB in the pregnant women and a high incidence of both miliary and cerebral TB was evident. With timely treatment, prognosis is positive.

## Introduction

Although a number of anti-tubercular drugs have been developed over the last 70 years, TB is still the leading cause of death from a single infection agent and, moreover, it is recognized as one of the top 10 causes of death^1^. In 2017, TB affected nearly 10 million people, 3.2 million of which were females; moreover, it was responsible for an estimated 1.6 million deaths globally^1^. Indeed, it is one of the main causes of death for women in their fertile years^2^. However, exact TB data with respect to pregnancy is currently unknown in China. Available numbers are based on the ratio of cases of women at the reproductive age combined with the crude birth rate in a particular area^3^. It has been estimated that, in 2011, there was many as 216,500 active TB cases in pregnant women globally^4^; moreover, the number of TB cases in pregnant women in the South-East Asia World Health Organization (WHO) Region was estimated to be 67,500^4^; finally, 9,500 active TB cases in pregnant women were estimated in China, which amounts to 0.7 per 1000 pregnant women and 4.4% of global burden among pregnant women^4^. Indeed, pregnancy-related TB represents a significant problem for both women and foetuses^5^. Ever since pregnancy-related TB was first discussed by David Stewart in 1922, it has been considered as an important cause of morbidity and mortality^6,7^. Some obstetricians have observed that the incidence rates of active TB are higher, and the progress more rapid, in pregnant cases compared to non-pregnant cases^7^. Unfortunately, the atypical clinical symptoms of TB (shortness of breath, fever, no weight gain, poor appetite and fatigue) mimic the physiological changes of pregnancy itself; moreover, physicians are largely reluctant to arrange the requisite examinations, such as a chest X-rays; therefore, diagnosing pregnancy-related TB is challenging^3,8^. In China, the national total TB incidence was about 1.41 million in 2017^1^; however, information on pregnancy-related TB is not readily available. In this study, we investigated the clinical characteristics of pregnant Chinese women with TB. The aim of the research is to explore the problems that exist in the diagnosis of TB during pregnancy, which, in turn, will promote awareness of TB diagnosis in pregnant women for clinicians.

## Results

The sample consisted of 28 women with TB during pregnancy and 28 non-pregnant women of fertile age with active TB; these cases were documented between May of 2012 and May of 2017 at Beijing Chest Hospital. The IQR of age in the pregnant women was 27–33, and the IQR of age in the non-pregnant women was 23–29. The human immunodeficiency virus antibody of the two groups was negative. They had no history of past TB, nor did they have any comorbid disease. The other baseline characteristics of the pregnant women and non-pregnant women are compared in Table 1. Indeed, the number of married pregnant women exceeded the number of non-pregnant women; the statistical difference was significant with P < 0.05. Moreover, between the two groups, the following elements did not reach statistical significance, since P > 0.05: previous treatments for TB, Bacillus Calmette-Guérin (BCG) vaccination status and contact history of TB. The basis on which TB diagnosis was made during pregnancy is given in Table 2. The symptoms of TB in pregnant women and non-pregnant women are shown in Table 3. The ratio of ‘poor appetite’ and ‘failure to gain weight’ was higher in pregnant women than in non-pregnant women, and the statistical difference was significant with P < 0.05. The ratio of ‘night sweating’ and ‘respiratory symptoms’ (cough, expectoration, shortness of breath, chest pain and hemoptysis) among non-pregnant individuals was higher than that of pregnant individuals; however, the difference did not reach statistical significance, since P > 0.05. The incident of ‘fever’ among pregnant women was higher than that among non-pregnant individuals, and the incidents of ‘fatigue’, ‘headache’ and ‘lumbago’ was the same between the two groups; however, statistical significance was not reached by any of said factors, with P > 0.05.

**Table 1:** Baseline characteristics of pregnant women and non-pregnant women.

Baseline characteristics (n = 28)(%)	Pregnant women (n = 28)(%)	Non-pregnant women	X^2^ value	P value
Marital status			16.555	**0.000**
Single	1 (3.6)	16(57.1)		
Married	27 (96.4)	12(42.9)		
Previous treatment for TB			0.878	0.352*
Yes	9(32.1)	4(14.3)		
No	19(67.9)	24(85.7)		
BCG vaccination status			0.530	0.469
Yes	25(89.3)	22(78.6)		
No	3(10.7)	6(21.4)		
Contact history of TB			0	1*
Yes	5(17.9)	5(17.9)		
No	23(82.1)	23(82.1)		

Categorical data was compared using the Chi-square test or Fisher’s exact test. Statistical significance set to *P* < 0.05 and was emphasized in bold. ^*^Fisher’s exact test.

**Table 2:** Site of TB disease and diagnosis basis of 28 TB patients during pregnancy.

Case	Site of TB disease	Diagnosis basis
1	Miliary, pleural and cerebral	Miliary shadowing on chest CT. Intracerebral tuberculomas on head MRI. Pleural exudatum, monocyte(MONO)% 82% and adenosine deaminase(ADA) 56.3 U/L. Positive blood T-SPOT.TB. Smear, culture and Xpert MTB/RIF were not done in sputum or pleural effusion.
2	Miliary, cerebral and meninges	Miliary shadowing on chest CT. Intracerebral tuberculomas on head MRI. Abnormal CSF. Positive blood T-SPOT.TB. Sputum smear and culture were negative, CSF smear and culture were not done, and Xpert MTB/RIF was not done in sputum or CSF.
3	Pleural	Pleural exudatum, MONO% 91% and ADA 62.2 U/L. Positive blood and pleural T-SPOT.TB. Smear, culture and Xpert MTB/RIF were not done in sputum or pleural effusion.
4	Pleural	Complained of fever and chest pains for 3 weeks. Less pleural effusion and no extraction. Positive blood T-SPOT.TB. Response to treatment. Smear, culture and Xpert MTB/RIF were not done in sputum or pleural effusion.
5	Pleural	Pleural exudatum, MONO% 66.3% and ADA 57.4 U/L. Positive blood T-SPOT.TB. Pleural effusion culture positive. Strain identification is *M. Tuberculosis* complex. Sputum smear and culture were negative.
6	Pulmonary and pleural	Lung infiltrate on chest CT. Pleural exudatum, MONO% 93.6% and ADA 41.9 U/L. Positive blood T-SPOT.TB. Smear, culture and Xpert MTB/RIF were negative in sputum and pleural effusion.
7	Pulmonary and pleural	Lung infiltrate on chest CT. Pleural exudatum, MONO% 98% and ADA 34 U/L. Positive blood T-SPOT.TB. Sputum culture positive. Strain identification is *M. Tuberculosis* complex. Sputum Xpert MTB/RIF positive. Smear, culture and Xpert MTB/RIF were not done in pleural effusion.
8	Pulmonary	Lung cavity on chest CT. Positive blood T-SPOT.TB. Broncho alveolar lavage fluid smear and culture positive. Strain identification is *M. Tuberculosis* complex. Smear, culture and Xpert MTB/RIF were not done in sputum.
9	Pleural	Complained of fever and short of breath for 2 weeks. Less pleural effusion and no extraction. Positive blood T-SPOT.TB. Response to treatment. Smear, culture and Xpert MTB/RIF were negative in sputum.
10	Pulmonary and pleural	Lung cavity on chest CT. Pleural exudatum, MONO% 93.6% and ADA 49.8 U/L. Positive blood T-SPOT.TB. Sputum smear and culture positive. Strain identification is *M. Tuberculosis* complex. Sputum Xpert MTB/RIF positive. Smear, culture and Xpert MTB/RIF were not done in pleural effusion.
11	Pulmonary	Lung cavity on chest CT. Positive blood T-SPOT.TB. Sputum smear and culture positive. Strain identification is *M. Tuberculosis* complex. Sputum Xpert MTB/RIF positive.
12	Lumbar vertebra	Psoas abscess on lumbar vertebra MRI. Pus extracted from psoas abscess Xpert MTB/RIF positive.
13	Pleural	Pleural exudatum, MONO% 97.3% and ADA 47 U/L. Positive blood T-SPOT.TB. Smear was negative in sputum and pleural effusion, and culture and Xpert MTB/RIF were not done.
14	Pulmonary and pleural	Lung infiltrate on chest CT. Pleural exudatum, MONO% 91.7% and ADA50.4 U/L. Positive blood T-SPOT.TB. Smear, culture and Xpert MTB/RIF were negative in sputum and pleural effusion.
15	Pulmonary and pleural	Lung infiltrate on chest CT. Pleural exudatum, MONO% 100% and ADA 60.7 U/L. Positive blood and pleural T-SPOT.TB. Smear, culture and Xpert MTB/RIF were negative in sputum and pleural effusion.
16	Miliary, cerebral, hilar and mediastinal lymph nodes	Miliary shadowing, hilar and mediastinal lymph nodes enlargement on chest CT. Intracerebral tuberculomas, encephalocoele and aqueduct cerebri eclasis on head MRI. Positive blood T-SPOT.TB. Smear, culture and Xpert MTB/RIF were negative in CSF, and smear, culture and Xpert MTB/RIF were not done in sputum.
17	Miliary, pleural, cerebral and meninge	Miliary shadowing on chest CT. Pleural exudatum, MONO% 98.6% and ADA 184.3 U/L. Intracerebral tuberculomas on head MRI. Positive blood T-SPOT.TB. Abnormal CSF. CSF smear was positive. Culture and Xpert MTB/RIF were negative in CSF. Xpert MTB/RIF was negative in pleural effusion. Smear, culture and Xpert MTB/RIF were not done in sputum.
18	Pulmonary, omentum majus, abdominal lymph nodes, liver and spleen	Lung infiltrate on chest CT. Abnormal abdomen enhanced CT. Granulomas in omentum biopsy. Positive blood T-SPOT.TB. Smear, culture and Xpert MTB/RIF were not done in sputum.
19	Pulmonary, hilar and mediastina lymph nodes	Lung cavity, hilar and mediastinal lymph nodes enlargement on chest CT. Bronchoscopy bronchial stenosis. Positive blood T-SPOT.TB. Sputum smear and Xpert MTB/RIF positive. Culture was negative in sputum.
20	Pulmonary and pleural	Lung infiltrate on chest CT. Pleural exudatum, MONO% 86.1% and ADA76U/L. Positive blood T-SPOT.TB. Smear and Xpert MTB/RIF was negative in pleural effusion, but culture was not done. Smear, culture and Xpert MTB/RIF were not done in sputum.
21	Miliary	Miliary shadowing on chest CT. Positive blood T-SPOT.TB. Smear, culture and Xpert MTB/RIF were not done in sputum.
22	Miliary and cerebral	Miliary shadowing on chest CT. Intracerebral tuberculomas on head MRI. Positive blood T-SPOT.TB. Smear, culture and Xpert MTB/RIF were not done in sputum.
23	Miliary, cerebral and meninges	Miliary shadowing on chest CT. Intracerebral tuberculomas on head MRI. Abnormal CSF. Response to treatment. Smear and Xpert MTB/RIF was negative in CSF, but culture was not done. Smear, culture and Xpert MTB/RIF were not done in sputum.
24	Pulmonary	Lung infiltrate on chest CT. Sputum culture positive. Strain identification is *M. Tuberculosis* complex. Positive blood T-SPOT.TB. Smear and Xpert MTB/RIF were not done in sputum.
25	Pulmonary	Complained of expectoration for 1 week. Lung infiltrate on chest CT. Positive blood T-SPOT.TB. Culture was negative in sputum, but smear and Xpert MTB/RIF were not done.
26	Pulmonary	Complained of cough and expectoration for 20 weeks. Lung infiltrate on chest CT. Positive blood T-SPOT.TB. Culture was negative in sputum, but smear and Xpert MTB/RIF were not done.
27	Pulmonary	Complained of cough and expectoration for 4 weeks. Lung infiltrate on chest CT. Positive blood T-SPOT.TB. Smear, culture and Xpert MTB/RIF were negative in sputum.
28	Miliary	Complained of fever and cough for 2 weeks. Miliary infiltrate on chest CT. Positive blood T-SPOT.TB. Culture was negative in sputum, but smear and Xpert MTB/RIF were not done.

**Table 3:** Symptoms of TB in pregnant women and non-pregnant women.

Symptoms	Pregnant women (n = 28) (%)	Non-pregnant women (n = 28) (%)	X^2^ value	P value
Fever	21(75.0)	19(67.9)	0.088	0.767
Night sweating	3(10.7)	7(25.0)	1.096	0.295
Fatigue	14(50.0)	14(50.0)	0	1*
Poor appetite	18 (64.3)	8(28.6)	5.815	**0.016**
Failure to gain weight	18(64.3)	7(25.0)	7.226	**0.007**
Cough	17(60.7)	19(67.9)	0.078	0.780
Expectoration	10(35.7)	14(50.0)	0.656	0.418
Shortness of breath	8(28.6)	12(42.9)	0.700	0.403
Chest pain	5(17.9)	9(32.1)	0.857	0.355
Headache	4(14.3)	4(14.3)	0	1*
Lumbago	1(3.6)	1(3.6)	0	1*
Hemoptysis	2(7.1)	6(21.4)	1.313	0.252^*^

Categorical data was compared using the Chi-square test or Fisher’s exact test. Statistical significance set to *P* < 0.05 and was emphasized in bold. ^*^Fisher’s exact test.

### State at time of diagnosis and duration of symptoms prior to diagnosis

Of the 28 women with TB during pregnancy, 15 (53.6%) were pregnant and TB was confirmed at a median of 14 weeks gestation (ranged 5–37 weeks); nine cases (32.1%) were confirmed within 42 days of delivery; TB was diagnosed at a median of four weeks among postpartum women (ranged 0–6 weeks). Moreover, four women (14.3%) had spontaneous abortions and the average abortion time was eight weeks of gestation, with TB diagnosed at a median of two weeks after abortion (ranged 1–4 weeks).

**Figure 1:**
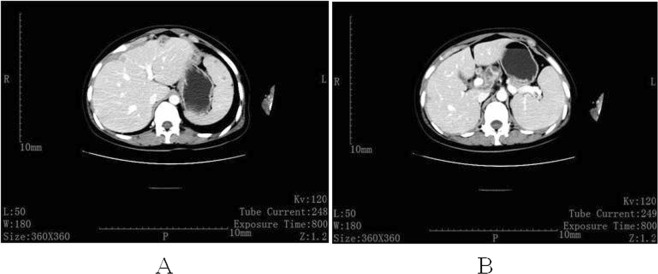
Abdominal enhanced CT of case 18. (**A**) Irregular thickness of the peritoneum and local nodular shape protruding to the liver surface; multiple nodules in the liver surface, with uneven enhancement and multiple local necrosis; larger in the spleen, with small circular low density and slight enhancement. (**B**) Multiple enlarged lymphoglandulae coeliacae coalescing partly and annular enhanced lesions, and low density shadow in the local liver.

Among the pregnant individuals, 14 patients (50%) did not have any X-ray or computerized tomography (CT) scans when they initially visited the hospital. Ten patients (35.7%) were initially misdiagnosed as having respiratory infections or pneumonia by obstetricians or physicians, all of whom subsequently underwent anti-infection treatment with penicillin or cephalosporin and/or azithromycin before TB was finally diagnosed. One patient (patient 18) presented with persistent uterine contractions at 28 weeks of gestation. Urgent caesarean section was performed at a local hospital, and an omental mass was found during the operation. The pathology of the mass revealed granulomatous inflammation, which was considered to be TB. Accordingly, anti-tubercular therapy (ATT) was initiated. Abdomen enhanced CT showed irregular thickness of the peritoneum, enhanced lesions in the liver and the spleen, and enlarged lymphoglandulae coeliacae (Fig. 1). The delay from the onset of symptoms to confirmed diagnosis in the remaining 27 cases (96.4%) ranged from one to 47 weeks, with a median of five weeks. Said delay can be broken down into two categories: patient-related delays and doctor-related delays, the former consisted of 27 cases (96.4%), and the latter consisted of 10 cases (35.7%) specific to misdiagnosis. The reasons for delay are discussed below.

Among the non-pregnant individuals, 11 (39.3%) visited hospital in a timely manner and were promptly diagnosed with TB. The remaining 17 (60.7%) experienced delays in TB diagnostics, ranging from one to 124 weeks with a median of four weeks. Moreover, 12 patients (42.9%) experienced delays due to their own fault, failing to visit a doctor in the appropriate time, and 11 (39.3%) were doctor-related caused by misdiagnosis. In six cases, both patient- and doctor-related delays were documented.

### Diagnosis and management

Among pregnant individuals and non-pregnant individuals, eight (28.6%) and 17 (60.7%) were diagnosed with definite TB, respectively, while 20 (71.4%) and 11 (39.3%) were considered to be probable TB, respectively. In the definite TB cases, three pregnant cases and five non-pregnant cases were confirmed by positive culture; the strain identification was *M. tuberculosis* complex; two pregnant cases and five non-pregnant cases were positively diagnosed using the Xpert MTB/RIF test; three pregnant cases and four non-pregnant cases were positively diagnosed by culture and the Xpert MTB/RIF test. Finally, among the non-pregnant cases, three cases were diagnosed by performing molecular pathology on either the lungs or the lumbar tissue.

Among pregnant individuals, 22 (78.6%) had PTB; 14 cases had coexisting PTB and EPTB; eight cases (28.6%) had disseminated miliary TB, six of which (21.4%) suffered from cerebral tuberculomas; three of these six also had meningitis involvement with abnormal cerebrospinal fluid (CSF), which is to say they had mononuclear pleocytosis with decreased levels of glucose and elevated proteins. Among pregnant individuals, moreover, six cases (21.4%) were exclusively EPTB, including five cases of tuberculous pleural effusion and one case with lumbar TB. All patients were treated with anti-TB drugs once they were diagnosed. ATT (isoniazid (H) 300 mg per day) was initially prescribed for 27 new patients, as was rifampicin (R) 450 mg per day, ethambutol (E) 750 mg per day and pyrazinamide (Z) 1500 mg per day. Six cases had cerebral and/or meningitis involvement; they were given an additional dose of moxifloxacin (400 mg per day), and the amount of H was adjusted to 500 mg per day. One patient (patient 18) had previously been treated with H, R, E, Z and levofloxacin (Lfx, 500 mg per day) for a period of two months, but the absorption of lung lesions was not obvious. She was treated with amikacin (0.4 g per day) on the basis of H, R, E, Z and Lfx.

### Outcomes following pregnancy-related TB

**Figure 2:**
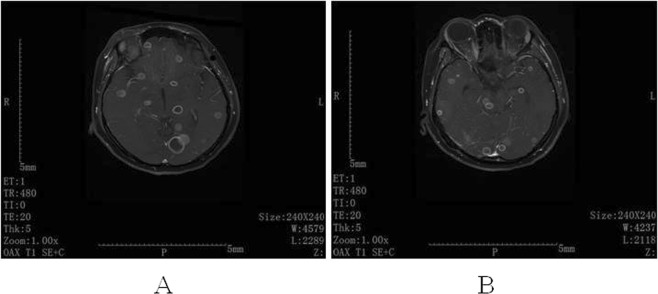
Cerebral enhanced MRI of case 17. (**A**) Multiple nodular and annular enhanced lesions in cerebrum and cerebellum. (**B**) Multiple nodular and annular enhanced lesions in cerebrum, cerebellum and brainstem.

At the end of treatment, 27 (96.4%) patients were cured. They were followed up for six months after ATT discontinuation; recurrences were not recorded. The remaining patient (patient 17) was initially misdiagnosed as having pneumonia at a local hospital. She was treated with cephalosporin and azithromycin for 11 weeks, which resulted in increasingly severe progression of the disease. She suffered from miliary TB and, after magnetic resonance imaging (MRI) of her head, multiple intracerebral tuberculomas were identified (Fig. 2). Moreover, the smear of CSF was positive for acid-fast bacilli. Unfortunately, she died during the treatment.

Four women had spontaneous abortions, which may be related to the maternal TB disease. Among the 15 pregnant women, nine selected to terminate the pregnancy; the others saw out pregnancy. Four neonates were healthy; one was SGA and one was neonatal TB. The remaining nine women were postpartum; five neonates were healthy; one was SGA and one was a dead fetus; two had neonatal TB, and one of them died at four months old.

## Discussion

TB remains a major worldwide public-health problem, posing a large threat to human health. It is the main cause of maternal death in the childbearing age and it is also a common non-obstetric cause of maternal mortality^9^. The accurate diagnosis of pregnancy-related TB was difficult, as the pregnancy symptoms overlapped with those of TB and other infectious and/or non-communicable diseases^9^. Generally speaking, TB in pregnant women is often atypical and difficult to diagnose in the early stages, which is problematic since diagnostic delays adversely affect treatment outcomes. According to WHO, three million active TB cases are missed annually; obviously, these require identification^10^. In this study, 28 inpatient cases of maternal TB over a five-year period in a hospital in China with a large TB incidence seemed relatively small, suggesting a large number of cases were missed.

In this study, 96.4% (27/28) cases were delayed due to the patients themselves, and 35.7% (10/28) were due to doctor error. Indeed, atypical onset of pregnancy-related TB may lead to diagnostic and treatment delays, which adversely effects pregnancy outcomes. Firstly, TB symptoms as poor appetite (18/28, 64.3%) and fatigue (14/28, 50%) are similar to the physiological symptoms of pregnancy. Moreover, normal weight gain during gestation may conceal the weight reduction caused by TB; 64.3% (18/28) of women failure to gain weight in this study. As a consequence, TB symptoms overlapped considerably with those of pregnancy. There was a larger number of incidents of patient-related delays in the pregnant group (96.4%) than in the non-pregnant group (42.9%). Additionally, the symptoms and signs of pregnancy-related TB, such as fever, coughing and expectoration, overlapped with respiratory infection and pneumonia. Indeed, the fact that the obstetricians and physicians were relatively unfamiliar with TB resulted in diagnostic delays or, worse still, misdiagnosis. Concerningly, 35.7% (10/28) of patients were misdiagnosed with having respiratory infection and/or pneumonia; they were treated with antibiotics before TB diagnostics. A review of medical records in London suggests that, in general, the median diagnosis delay is 32 days^11^. In this study, however, it was five weeks.

Not surprisingly, TB symptoms during pregnancy were less common than postpartum, which was probably due to changes in immunity and physiology^12^. After all, according to Zenner, *et al*., the incidence of TB six months after delivery is higher than it is during pregnancy^13^. Moreover, a cross-sectional survey conducted on both Europe and the US suggests that TB diagnosis is made more frequently three months after delivery than it is during pregnancy^14^. In this study, 42 days after delivery was selected as the cut-off point for pregnancy-related TB. This was because, after 42 days, the physiological changes associated with pregnancy typically revert back to normal^15^. Secondly, the clinicians made their diagnoses during puerperium if the TB cases were within 42 days of delivery; however, they would not make special pregnant or puerperium diagnosis for TB cases past 42 days of delivery. Finally, the coders would not encode the disease; therefore, we could not research TB cases over 42 days postpartum.

In the study, 50% (14/28) of patients did not have any chest X-rays or CT scans upon visiting local hospitals, which resulted in diagnostic delays. According to the most recent guidelines, X-rays and CT scans are safe during pregnancy and lactation^16^. Therefore, they should be encouraged in the diagnosis of pregnancy-related TB; the potential risk to the fetus can be minimised by abdominal shield. Overall, the nonspecific symptomatology, reluctance for imaging tests, as well as a general lack of awareness are possibly related to diagnostic and treatment delays, which, in turn, can result in poor prognosis in general for mothers and neonates^17^.

Pregnancy-related TB is relevant since the mortality rates are high for both mothers and neonates during pregnancy and postpartum^7^. In this study, 28.6% (8/28) of women were diagnosed with miliary TB. Simultaneously, 21.4% (6/28) were diagnosed with cerebral TB. Unfortunately, one mother died. In total, 15 neonates were born, nine of which were healthy; the remaining six had neonatal complications associated with maternal TB; unfortunately, two died. An analysis in western Kenya documented that 10% of pregnant-women deaths are caused by TB^18^. One post-mortem research on pregnancy-related death has been conducted in Africa, identifying TB as the cause of death in 12.9% of mothers^19^. Indeed, TB can be transmitted through the dissemination of blood during pregnancy, the inhalation of amniotic fluid during delivery, and by virtue of postnatal respiratory droplets^20^. TB increased the perinatal mortality by a factor of six and it increased the risk of premature delivery as well as small birth weight by a factor of two^21^. A study of infant outcomes in TB-infected mothers in Cape Town suggests that 65% were premature and 59% had small birth weights^22^. Indeed, integration of TB screening in pregnant women may improve maternal and neonatal outcomes^23^.

Obviously, TB among pregnant women has public-health importance not only because of the risk of mortality and morbidity to the mothers, but also because it is harmful to the neonates^24^. Therefore, once pregnant women are diagnosed with active TB, action should be taken immediately^8^. According to WHO, TB during pregnancy should be treated with four first-line drugs (H, R, E and Z) for the first two months and then with another two drugs (H and R) for the next four months^25^. If patients are sensitive to anti-TB drugs and have good adherence, roughly 90% can be cured^26^. In this study, all patients were treated with the standard treatment regimen once TB was diagnosed; they had good adherence with 96.4% (27/28) of patients cured after anti-TB treatment, while only 3.6% (1/28) died. Therefore, timely anti-TB treatment is vital for curing TB.

This study is not without its limitations. Firstly, the sample size was quite small. Beijing Chest Hospital is one of the referral TB hospitals in China and the majority of patients were from Beijing or adjacent provinces. Indeed, if the sample size is expanded and diversified, then pregnancy-related TB will be comprehensively represented. Another limitation was that only 28.6% of patients were diagnosed by bacteriological examination, while 20 patients (71.4%) were diagnosed clinically.

## Materials and Methods

### Study design

The study was approved by the Ethics Committee of Beijing Chest Hospital, Capital Medical University. Written consent was waived as this was a retrospective investigation. All records were anonymous and confidential. This was an evaluation of all TB hospitalized cases during pregnancy from May 2012 to May 2017, at Beijing Chest Hospital. TB cases were identified based on the diagnosis codes. The coders encoded the TB disease, pregnancy, childbirth and the puerperium according to the international Classification of diseases (ICD-10). Patient was considered to infect with TB during pregnancy if the diagnosis was made during pregnancy and postpartum, and, moreover, if TB symptoms clearly began to manifest during pregnancy. To ensure that TB cases were not omitted during pregnancy, patients diagnosed with TB from pregnancy until 42 days after delivery were included. Women who suffered from TB before pregnancy and conceived during the period of ATT were excluded from this study. We matched the TB cases during pregnancy with age, place of residence, comorbid disease, time of hospitalization and the site of TB disease in the selection of the control group (Supplementary Dataset 1 and 2). We used 28 non-pregnant women of fertile age (age 20–40) with active TB as the control group; they were hospitalized from May 2012 to May 2017. Most of them were also from Beijing or adjacent provinces. The site of TB disease was the same with pregnant individuals. Information such as demographic, medical history, clinical symptom, radiographic features, laboratory-test results, therapeutic regimens, and the outcomes of pregnant women and neonates were available in the medical records. A follow-up phone call was conducted with the participants. Diagnostic delays were defined as the time elapsed from the initial onset of symptoms recorded in the patients’ medical charts to the final TB diagnosis. Patient-related delays were defined as the interval from symptom onset to the time at which medical attention was sought, while doctor-related delays were defined as the time from patients first attending hospital to the definitive diagnosis of TB.

### Diagnostic criteria and classification of TB

A patient was diagnosed as having definite TB based on the identification of *M. tuberculosis* complex in the clinical sample (sputum, body fluid, or body tissue), either by culture or molecular method^27^. The Xpert MTB/RIF test (a molecular method) was used to rapidly detect the diagnosis of TB, as recommended by WHO^1^. A diagnosis of TB was considered to be probable, and based on clinical, radiographic evidence and the clinician prescribed a full course of ATT.

In accordance with the WHO definitions for TB, PTB was defined as TB involving lung parenchyma, which included miliary TB; EPTB referred to the presence of *M. tuberculosis* in organs, except the lung. A patient with both PTB and EPTB was classified as PTB^27^. New patients were defined as those who had never received TB treatment or taking ATT within one month^27^. Previously treated patients were those who, in the past, had taken ATT for more than one month^27^.

### Data analysis

The medical records were analyzed, with descriptive analysis conducted for clinical data. For statistical analysis, R language version 3.4.4 was used. The results were presented in terms of IQR according to age, as well as in terms of medians and percentages with respect to the categorical variables. Categorical data was compared using the Chi-square test or Fisher’s exact test. All P-values were calculated with statistical significance set to P < 0.05.

## Supplementary information

Supplementary Information

Dataset 1

Dataset 2

## References

[1] World Health Organization. Global Tuberculosis Report 2018. Geneva: World Health Organization, (2018).

[2] Say L (2014). Global causes of maternal death: a WHO systematic analysis. Lancet Glob Health..

[3] Sulis G, Pai M (2018). Tuberculosis in Pregnancy: A Treacherous Yet Neglected Issue. J Obstet Gynaecol Can..

[4] Sugarman J, Colvin C, Moran AC, Oxlade O (2014). Tuberculosis in pregnancy: an estimate of the global burden of disease. Lancet Glob Health..

[5] El-Messidi A, Czuzoj-Shulman N, Spence AR, Abenhaim HA (2016). Medical and obstetric outcomes among pregnant women with tuberculosis: a population-based study of 7.8 million births. Am J Obstet Gynecol..

[6] Stewart DA (1922). Pregnancy and tuberculosis: Part I-The effects of pregnancy on tuberculosis. Can Med Assoc..

[7] Bates M (2015). Perspectives on tuberculosis in pregnancy. Int J Infect Dis..

[8] Gould, J. M. & Aronoff, S. C. Tuberculosis and pregnancy-maternal, fetal, and neonatal considerations. *Microbiol Spectr*. **4**, (2016). 10.1128/microbiolspec.TNMI7-0016-2016 28084207

[9] Zumla A, Bates M, Mwaba P (2014). The neglected global burden of tuberculosis in pregnancy. Lancet Glob Health..

[10] Herbert N (2014). World TB Day 2014: finding the missing 3 million. Lancet..

[11] Kothari A, Mahadevan N, Girling J (2006). Tuberculosis and pregnancy-results of a study in a high prevalence area in London. Eur J Obstet Gynecol Reprod Biol..

[12] Singh N, Perfect JR (2007). Immune reconstitution syndrome and exacerbation of infections after pregnancy. Clin Infect Dis..

[13] Zenner D, Kruijshaar ME, Andrews N, Abubakar I (2012). Risk of tuberculosis in pregnancy: a national, primary care-based cohort and self-controlled case series study. Am J Respir Crit Care Med..

[14] Bothamley GH (2016). Pregnancy in patients with tuberculosis: a TBNET cross-sectional survey. BMC Pregnancy Childbirth..

[15] Hoyert DL (2007). Maternal mortality and related concepts. Vital Health Stat 3..

[16] Committee on Obstetric Practice. (2017). Committee opinion No. 723: guidelines for diagnostic imaging during pregnancy and lactation. Obstet Gynecol..

[17] Zenner D, Ashkin D (2016). Diagnosis of latent tuberculosis infection in HIV-infected pregnant women. “baby steps” toward better tuberculosis control in pregnancy. Am J Respir Crit Care Med..

[18] Desai M (2013). An analysis of pregnancy-related mortality in the KEMRI/CDC health and demographic surveillance system in western Kenya. PLoS One..

[19] Menendez C (2008). An autopsy study of maternal mortality in Mozambique: the contribution of infectious diseases. PLoS Med..

[20] Mathad JS, Gupta A (2012). Tuberculosis in pregnant and postpartum women: epidemiology, management, and research gaps. Clin Infect Dis..

[21] Bishara H (2015). Tuberculosis during pregnancy in Northern Israel, 2002-2012: epidemiology and clinical practices. Isr Med Assoc J..

[22] Bekker A, Schaaf HS, Draper HR, Kriel M, Hesseling AC (2016). Tuberculosis disease during pregnancy and treatment outcomes in HIV-Infected and uninfected women at a referral hospital in Cape Town. PLoS One..

[23] Cranmer LM (2017). Integrating tuberculosis screening in Kenyan prevention of mother-to-child transmission programs. Int J Tuberc Lung Dis..

[24] Gebreegziabiher D, Adane K, Abebe M (2017). A survey on undiagnosed active pulmonary tuberculosis among pregnant mothers in Mekelle and surrounding districts in Tigray, Ethiopia. Int J Mycobacteriol..

[25] World Health Organization. Treatment of tuberculosis: guidelines - 4th ed (ed. World Health Organization) 29–51 (World Health Organization Press, 2010).

[26] Loto OM, Awowole I (2012). Tuberculosis in pregnancy: a review. J Pregnancy..

[27] World Health Organization. Treatment of tuberculosis: guidelines - 4th ed (ed. World Health Organization) 23–28 (World Health Organization Press, 2010).

