# Supplementary material for: Retrospective Analysis of 28 Cases of Tuberculosis in Pregnant Women in China
Source: Sci Rep. 2019 Oct 25;9:15347. doi: 10.1038/s41598-019-51695-8 (PMC6814810; doi:10.1038/s41598-019-51695-8)
Supplement: Supplementary file 1 — Supplementary Information [file 41598_2019_51695_MOESM1_ESM.pdf]

Retrospective Analysis of 28 Cases of Tuberculosis in Pregnant Women in China

Qiang Li[1], Yanhua Song[1], Hongmei Chen[1], Li Xie[1], Mengqiu Gao[1]*, Liping Ma[1]*, and Yinxia Huang[2]*

[1]Department of Tuberculosis, Beijing Chest Hospital, Capital Medical University & Beijing Tuberculosis and Thoracic Tumor Research Institute, Beijing, China; [2]Beijing Key Laboratory for Drug Resistant Tuberculosis Research, Beijing Tuberculosis and Thoracic Tumor Research Institute, Beijing Chest Hospital, Capital Medical University, Beijing, China. Correspondence and requests for materials should be addressed to M.G.(email:gaomqwdm@aliyun.com), L.M.(email:malipinghm@hotmail.com) or Y .H.(email:huangxx2001@sina.com)
